# Supplementary material for: MetaRibo-Seq measures translation in microbiomes
Source: Nat Commun. 2020 Jun 29;11:3268. doi: 10.1038/s41467-020-17081-z (PMC7324362; doi:10.1038/s41467-020-17081-z)
Supplement: Supplementary file 10 — Supplementary Data 7 [file 41467_2020_17081_MOESM10_ESM.zip › File2/Confidence_High_Taxonomy/305460_out.krona.html]

Javascript must be enabled to view this page.

members
magnitude
magnitudeUnassigned
count
unassigned
taxon
rank

305460\_out

7

7
2
superkingdom

1239
phylum
7

class
186801
7

order
186802
7

7
family
186803

genus
33042
2

SRS078242\_contig\_number\_17550SRS1041145\_contig\_number\_36764
7

species

SRS011084\_contig\_number\_34211SRS015431\_contig\_number\_46332SRS104400\_contig\_number\_7220
2049024
3

2
33043

SRS019068\_contig\_number\_110465SRS047014\_contig\_number\_31082
species
